# VR-CARE: a protocol for a mixed-methods study and pilot trial with embedded process evaluation to develop and evaluate virtual reality training for risk reduction in care homes

**DOI:** 10.1136/bmjopen-2026-116603

**Published:** 2026-03-09

**Authors:** Norina Gasteiger, Claire R Ford, Helen Hawley-Hague, Jack Wilkinson, Debra Jones, William Whittaker, Akbar Ullah, Roman Kislov, Emma Stanmore, Louise Laverty, Joan Chantrell, Claire Callaghan, Victoria Edmondson, Dawn Dowding

**Affiliations:** 1 Division of Nursing, Midwifery and Social Work, The University of Manchester, Manchester, UK; 2 Centre for Biostatistics, Manchester Academic Health Science Centre, Division of Population Health, Health Services Research, and Primary Care, The University of Manchester, Manchester, UK; 3 University of Liverpool, Liverpool, UK; 4 Division of Population Health, Health Services Research, and Primary Care, The University of Manchester, Manchester, UK; 5 Centre for Decent Work and Productivity, Manchester Metropolitan University, Manchester, UK; 6 The University of Manchester, Manchester, UK; 7 School of Dentistry, Faculty of Medicine and Health, University of Leeds, Leeds, UK; 8 Public Contributor, The University of Manchester, Manchester, UK; 9 Care Home Representative, The University of Manchester, Manchester, UK

**Keywords:** Virtual Reality, Nursing Homes, Hand Hygiene, Capacity Building, EDUCATION & TRAINING (see Medical Education & Training)

## Abstract

**Abstract:**

**Introduction:**

Risk reduction training for UK care home staff is limited, not standardised and challenging to implement. Virtual reality (VR) is an immersive, engaging method of education delivery that is being adopted in health and social care. VR may be an effective education tool in care homes, but this research has yet to be conducted.

The VR-CARE project aims to create a new VR risk reduction training programme for care homes that combines hand hygiene and falls prevention modules, and to evaluate this through a pilot trial to inform a future randomised controlled trial (RCT).

**Methods and analysis:**

There are two research phases with patient and public involvement and engagement (PPIE) activities embedded throughout. Care home stakeholders are collaborating to design the training and toolkit, oversee methods, review resources for accessibility, support recruitment and ensure the project meets the needs of the workforce and positively impacts resident care.

In phase 1, we will use a mixed-methods and user-centred design approach to develop the VR training and an accompanying implementation toolkit needed to deliver it. The training will be developed and tested by 15 care home staff across three rounds to identify and inform changes that maximise usability and acceptability. We will conduct up to 20 interviews with staff from VR companies and care homes to support toolkit development.

Phase 2 is a mixed-methods pilot cluster RCT, with a waitlist control and process evaluation with up to 80 unregistered staff members from six North England care homes, to develop the measures and methods to inform a future trial. The process evaluation will generate knowledge about VR as a training mechanism in care homes. This phase will focus on the practicality of using VR, broader impacts (eg, on residents), contextual considerations and how it might be scaled up.

**Ethics and dissemination:**

The University of Manchester Proportionate University Research Ethics Committee has approved phase 1 (Reference: 2025-24416-44642). We will obtain further approval before commencing phase 2.

Outputs will include user-friendly and acceptable VR risk reduction training for care homes, accompanied by an implementation toolkit adaptable for other VR training in social care settings. Materials (eg, training overviews, infographics and videos) will be developed to support uptake. Findings will be presented at conferences and published in journals. Lay summaries will be co-created with our PPIE group, and additional dissemination methods will be co-developed to broaden reach.

**STRENGTHS AND LIMITATIONS OF THIS STUDY**

- The mixed-methods approach enables a comprehensive understanding of several components that may impact the success of the training in the future, including iterative development, usability testing, qualitative understandings of implementation experiences, cost implications and measured impacts on both residents and staff.
- This project will help develop the measures and methods to inform a future randomised controlled trial.
- Inherent technical barriers, such as cybersickness, digital literacy (and confidence) and internet access in care homes may limit engagement, satisfaction or fidelity of delivery.
- The 3-month follow-up time point will allow for an exploration of any sustained behavioural outcomes (eg, skill) and changes in practice or impacts on residents.
- The limited geographic scope may affect transferability to other areas and countries, as regional workforce characteristics, organisational cultures and digital infrastructure specific to the North of England may be reflected.

## Introduction

 In England, around 324 000 older adults live in long-term residential or nursing care facilities (hereafter referred to as care homes).^1^ Care home residents have complex health and care needs and, as such, are more prone to falls and infections. Residents are more exposed to infections due to the shared living environment,^2^ and 50% of residents fall each year.^3^ It is therefore essential to ensure that care home staff receive training to provide high-quality care. However, training in hand hygiene and falls prevention in care homes is not mandatory despite high rates of infectious disease outbreaks^4^ and falls.^5^

There are several challenges associated with delivering staff training in care homes. First, most care home staff caring for residents are unregistered healthcare workers. The King’s Fund^6^ reports that in 2024, there were 860 000 care workers and 83 000 senior care workers working in adult social care in England (which includes those being cared for in their own homes) compared with 33 000 registered nurses. These workers do not receive equal training opportunities compared with their registered counterparts (nurses), which affects how knowledge is applied in practice.^7^ Barriers to improving work practices include lack of staff knowledge, time and resources (ie, training).^8^ There are also training accessibility issues for staff working unsociable hours. High staff turnover leads to increased induction training, which is costly to care homes. Current training methods exhibit varying levels of effectiveness,^9 10^ and access to ongoing training for unregistered care workers is influenced by workload, workforce availability, staffing levels, cost and the prevalence of eLearning as the default training method.^9^

Challenges faced by staff impact their ability to care for residents, including unclear career development, heavy workloads^11^ and high vacancy rates, with 152 000 unfilled positions in adult social care.^12^ The health and social care turnover rate was 28.3% in 2022/2023, representing 390 000 leavers,^12^ identifying a staff retention issue. Investing in developing care staff through training can help improve staff retention^13^ and support the delivery of high-quality care.^14^ Care staff training can improve resident outcomes.^15^ Investing in education can also build confidence, preparedness, empower staff and improve organisational culture and workforce retention,^16 17^ reducing vacancy advertising and agency staff costs.

The adult social care setting is already increasingly adopting and accepting innovative technology, as reflected by a report published by the Department of Health and Social Care in 2021.^18^ Notably, 20% of social care organisations now consider themselves to be digitally ‘expert’ compared with 12% in 2019, and organisations are favouring digital solutions, ranging from sensors, alarms and telehealth to robotics and artificial intelligence.^18^ Additionally, it is stated that most applications of digital technology have been for workforce learning rather than resident-facing care.

Virtual reality (VR), an immersive three-dimensional simulated environment where users can interact with equipment such as a headset, is beginning to be used in social care, including for reminiscence/distraction therapy for residents and for staff training. VR can meet care home staff training needs as it is faster, practical, cost-saving and increases knowledge transfer in comparison to usual training delivery methods.^19 20^

We have already conducted two successful programmes of work exploring the usability and acceptability of VR for training care home workers in hand hygiene and falls prevention, which received positive responses from staff.^21^,^23^ One of the studies developed the VR training in direct response to requests from care home staff, indicating a desire for more innovative approaches to training in the sector. There is scope to combine these topics into a risk reduction training package and expand to other topics in the future. For example, other research has reported on VR dementia awareness training in UK care homes.^24^

In our previous work, collaborating with care home staff to develop training, the specific approach requested and subsequently developed by us was immersive training in falls prevention through VR. Additionally, in our multi-site study with five care homes in North England, we found that immersive VR training for hand hygiene was more acceptable to care staff than the non-immersive computer-based version, with the highest knowledge improvements also made by the group using immersive VR training.^23^

Systematic reviews with meta-analysis have also explored the effectiveness of VR training for health workers, reporting greater efficacy in improving knowledge and skills than non-immersive training.^25 26^ Our realist synthesis of 80 papers also found that immersion, interactivity, learning in a safe environment, repeated practice and the realism of VR are conducive to learning skills and improving knowledge.^27^ Identified barriers in the review and other research include negative attitudes, cybersickness (VR-induced nausea), logistics, upfront costs and training content development.^27 28^ As also highlighted in the review, the cost-effectiveness of VR training has facilitated implementation in other settings. This is because VR training can be used autonomously and repeatedly, with no additional cost per learner. It can also serve as a comprehensive training tool used on-site, thereby reducing travel for educators and eliminating the need for additional training resources. However, none of the studies included in the review were conducted in care home settings.

The effectiveness, benefits to residents and cost-delivery models of VR training for care home staff have not yet been evaluated; as such, its impact and how to support implementation remain unclear. Research is also needed to assess setup and maintenance costs.^25^ A more comprehensive understanding of feasibility, acceptability, usability, impact and economic implications is required to enhance the overall success and scale-up of VR training in care homes across the UK.

This study builds on our work with care homes across Greater Manchester, providing insights into how VR training for unregistered staff can be integrated into current training packages and the potential impacts on the workforce and residents. It will further develop the VR training packages, expanding beyond Greater Manchester to explore its potential benefits across the care home sector. Given the lack of evidence in this field, the study proposes to explore the feasibility of robustly evaluating VR in care homes through a randomised controlled trial (RCT).

### Aims and objectives

This project will inform a wider programme of work that seeks to explore whether VR risk reduction training (focusing on hand hygiene and falls) by care home staff increases their knowledge, skills and job satisfaction, improves staff retention, reduces infections and falls among residents and whether it is a cost-effective use of NHS and social care resources.

We aim to use the principles of the existing VR falls and hand hygiene training by combining both modules to create a new VR risk reduction programme for care homes, and conduct a pilot trial to inform a future RCT.

The objectives are to:

1. Develop, refine and understand the requirements of a risk reduction VR training programme on hand hygiene and falls prevention, and an accompanying implementation toolkit.
2. Explore the feasibility, acceptability and usability of the VR training in care homes.
3. Generate knowledge about VR as a training mechanism in care homes and consolidate and present our knowledge alongside an implementation toolkit relevant to other VR training programmes in similar social care settings.
4. Develop the methods to inform an RCT to assess effectiveness and impact, staff satisfaction and retention, together with the economic implications and implementation of the VR risk reduction training programme in care homes.

## Methods and study design

### Team

The project is being conducted by a team with expertise in developing VR training for care staff, evaluating digital interventions (including VR), qualitative and mixed-methods research, implementation science, economic evaluations, statistical analyses and trials, educational pedagogies and technology commercialisation. A group of care home staff and visitors collaborate on the project to ensure it is meaningful (see the Patient and public involvement and engagement section). An external oversight (steering) committee has also been established to monitor the project and will convene at least once annually.

### Study design

**Figure 1:**
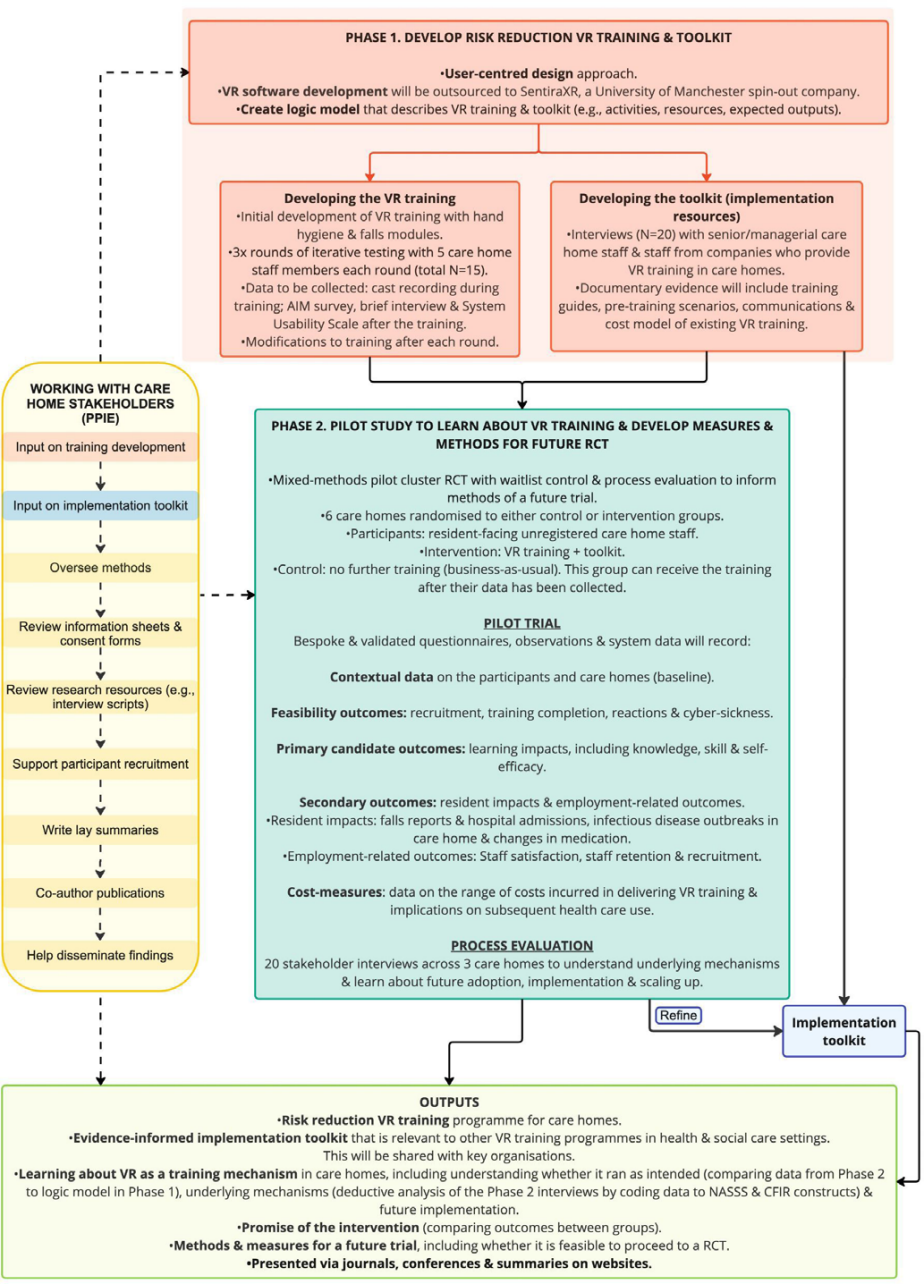
Flow diagram portraying the key activities of the VR-CARE project. AIM, 4-item Acceptability of Intervention Measure; CFIR, Consolidated Framework of Implementation Research; NASSS, Nonadoption, Abandonment, Scale-up, Spread and Sustainability; PPIE, patient and public involvement and engagement; RCT, randomised controlled trial; VR, virtual reality.

This project consists of two phases conducted from September 2026 to March 2028: (a) developing, testing and refining the VR risk reduction training and implementation toolkit and (b) pilot study with process evaluation to explore the VR training and develop the measures and methods to inform a future RCT. Figure 1 presents the project activities, indicating that collaboration with care home stakeholders will be embedded throughout the project.

Both phases require primary data collection through interviews, questionnaires/surveys, observations and the collection of data related to the costs of the training intervention and its impact on resident health outcomes. Each phase will be guided by the MRC Framework for Developing Complex Interventions^29^ to ensure that context, stakeholder engagement, uncertainties and economic considerations are explored. Developing and refining the implementation toolkit is a continuous thread that runs through each phase of the project. The Standards for Reporting Implementation Studies Statement^30^ and the Consolidated Standards of Reporting Trials extension for reporting randomised pilot studies^31^ will be used.

As part of phase 2, we will undertake a qualitative process evaluation informed by the Nonadoption, Abandonment, Scale-up, Spread and Sustainability (NASSS) framework^32^ and the Consolidated Framework of Implementation Research (CFIR),^33^ whereby participant interviews will be conducted to help understand the underlying mechanisms, pilot trial outcomes and explore how the VR training might be adopted, implemented and scaled up.

In each data collection phase, we will gather protected demographic information for care home staff, including age, gender and ethnicity, in accordance with National Institute for Health and Care Research (NIHR) guidelines. Additionally, we will collect employment-related data, such as length of employment and job role/title, which will be aggregated to generate participant summaries and ensure that our recruitment process promotes inclusivity. We have also conducted an Equality Impact Assessment and will consider this throughout our project (see table 1).

**Table 1:** Equality impact assessment for the VR training and associated research activities

Characteristics	Is there a potential for positive or negative impact?	Explanation	Action to address negative impact
Disability	Potentially negative	Potential challenges in engaging with the training may arise when experiencing mild hearing/visual impairment, mobility issues or neuro-disabilities	Users can stop, pause, restart or resume the training at any point to take breaks if and when required The headset is adjustable for varying head sizes and can be worn with spectacles The headset volume can be adjusted, and written text will guide learners through the scenarios. There will also be audio to support the written text The training can be completed in a stationary position, as well as in a standing or sitting position The user can use one handset in their dominant hand to interact with the scenarios The VR scenarios will be interactive and guide users, making them more appropriate for people with learning disabilities
Gender reassignment	No impact	–	–
Marriage or civil partnership	No impact	–	–
Pregnancy and maternity	Potentially negative	VR is generally safe for pregnant individuals and has been successfully used to reduce anxiety and prepare women for labour. However, some women may experience hesitancy, nausea or balance concerns when using VR	The training can be completed sitting down The Participant Information Sheet will suggest that concerned individuals consult their GP to confirm it is safe for them to take part in the training
Race	No impact	–	–
Religion or belief	No impact	–	Where possible, training and data collection will not take place during religious or cultural holidays Headsets are adjustable to fit varying head sizes or any religious headwear
Sexual orientation	No impact	–	–
Sex (gender)	No impact	–	–
Age	No impact	–	–
Other: caring responsibilities	No impact	–	Training will be available at the workplace of participants and therefore will not interfere with any caring responsibilities or dependents the staff may have
Other: technology experience	Potentially negative	People using VR for the first time may be unfamiliar with navigating immersive environments. They may lack confidence in using the training	An ‘onboarding’ session will be provided as part of the training to guide users on how to use VR All training materials will be accessible and aimed at individuals with limited technology experience We will include photographs and a description of VR in Participant Information Sheets
Other: literacy levels	Potentially negative	Some care home staff may be non-native English speakers or have low literacy levels	We will develop the written text in the training and all research materials using the Flesch-Kincaid readability metrics to ensure that lay language is used We will provide audio to support any text in the training

GP, general practitioner; VR, virtual reality.

### Phase 1: developing the VR risk reduction training and implementation toolkit

The first phase comprises two research components: one dedicated to developing the VR training (a mixed-methods study) and the other to developing the implementation toolkit required to deliver it (a qualitative interview study). It addresses objectives 1, 2 and 4.

#### Developing the VR training

We will first develop a proof-of-concept of the VR training and then test and refine it with care home staff members across three testing rounds. Feedback (eg, on usability and acceptability) will be gathered during each round, with iterations to the training made in between. This iterative process will help create an intervention that prioritises user experience.

We will develop the risk reduction VR training programme with Sentira^XR^, a spin-out company based at the University of Manchester. The programme will host two modules (falls and hand hygiene) and will build on our previous work by using the same training principles.^21 23^ Combining the modules into a new programme is essential to improve user experience, as it will ensure that navigation is consistent. It also provides the potential to introduce other training modules in the future. Core elements include resident-focused care scenarios where learners identify opportunities to implement risk reduction techniques (eg, how and when to use hand hygiene and identify fall risk factors). This is underpinned by Virtual Andragogy,^34^ using multiple adult learning theories in technology.

##### Setting, participants and recruitment

This work will be conducted during work hours on-site at 4–6 care homes across Greater Manchester and the Northwest region (including nursing or residential homes). The care homes will be identified from our existing network of care homes that have expressed interest in participating. We will sample care homes by type (nursing, residential or mixed), size (number of beds), ownership (independent, chain, private, charity or public) and location (deprivation).

Participants will be included if they are unregistered staff members employed at the care home, providing direct care to residents, read/write in English and do not have previous health conditions that render them ineligible to use a VR headset due to safety reasons (eg, history of seizures, vestibular or neurological conditions). Staff who are registered (eg, nurses), unable to read/write in English or who do not provide care (eg, domestic staff or chefs) will be excluded.

We will recruit a total of 15 participants (up to three per home), with five participants in each round. This sample size has been deemed acceptable in user experience research, as five participants can identify 80% of a product’s usability issues.^35 36^

Purposive recruitment methods will be used, although we will employ maximum variation sampling to maximise diversity in the sample, considering age, gender, ethnicity and experience with VR, as well as working in care homes.

Managers of care homes will be asked to identify eligible care staff and provide them with a participant information sheet. This will state that participation is voluntary and that non-participation will not impact their employment. Staff will have a minimum of 24 hours to decide whether to take part and will be asked to contact the researchers if they are willing to participate in the study. The research team will collaborate with managers and consenting staff to schedule data collection dates, during which they will conduct sessions with individual participants.

##### Procedures

Participants will first provide written consent to participate and will be asked whether they would like to share their contact information (email address) to receive updates on the project, such as lay summaries. They will then complete a demographics questionnaire and a VR pre-screening form to assess whether it is safe for them to participate.

During each of the three rounds, we will observe participants using the training through a cast recording (visual capture of what the user sees through the VR headset) to identify any issues. Observations will also be recorded by the researchers using written notes. Participants will then complete the validated and reliable 4-item Acceptability of Intervention Measure (AIM) survey,^37^ the validated 10-item System Usability Scale (SUS)^38^ and the existing 6-item Cybersickness in VR Questionnaire (CSQ-VR).^39 40^ The CSQ-VR was chosen over others as it has superior psychometric properties, is a valid assessment of the most important cybersickness symptoms and is short to administer. We will also use different VR headsets throughout the rounds to explore how to minimise cybersickness.

A short semi-structured interview will follow, using survey responses as prompts, challenges in using the training, what support is needed and asking how the intervention and for round three, how the toolkit could be improved. This will be audio-recorded and transcribed verbatim for analysis.

Each session will be no longer than 1 hour to minimise any impact on work. All participants will receive a £20 voucher.

##### Analysis

The AIM, SUS and CSQ-VR questionnaire responses will be analysed using IBM SPSS, as per guidance from the respective questionnaire developers.

The Likert scale responses for the AIM questionnaire will range from 1 to 5, with a mean score generated across the items.^37^ There are currently no established cut-offs for interpretation; however, higher scores indicate better acceptability.

For the SUS, the Likert scale responses will range from 0 to 4, with higher scores representing more positive responses. Scores for the 10 items will then be summed and multiplied by 2.5 to give a total score of 100. The following interpretations will be used: <50 indicates poor usability, 70+ indicates good usability and 85+ indicates excellent usability.^41^

A total score and sub-scores for the nausea, disorientation and oculomotor symptoms will be produced for the CSQ-VR.^39 40^ The sub-scores will be calculated by adding the responses (on a Likert scale of 1–7) for the two questions related to each symptom. The total score is the sum of the sub-scores.

Observations and interview data will be managed on NVivo. Thematic analysis will be used due to its utility for multidisciplinary work and alignment with integrating multiple data sources.^42^ Integrating different data sources will be considered carefully to ensure equal weighting to the findings. The four steps to integration proposed by Cronin *et al*^43^ will be followed: (a) analysing each dataset and identifying tentative topics, (b) using the topics as threads for further analysis of the other datasets, (c) codes, categories and themes concerning the threads are used to create a data repository, (d) synthesis of the threads that incorporates the similarities and differences in the sources. At least two researchers will separately code the data, with initial themes sense-checked by the wider team and the patient and public involvement and engagement (PPIE) group.

### Developing the implementation toolkit

The accompanying toolkit aims to minimise barriers to use and optimise the implementation of the VR training. We will integrate the findings from the development of the VR training package with data collected from 20 interviews with senior care home staff who have experience with VR training and staff from companies that provide VR training. This will inform discussions with our PPIE group to develop the toolkit. We will also create a logic model of the training and test assumptions of potential accompanying tools and resources with the PPIE group to finalise the delivery method.

#### Setting, participants and recruitment

Up to 20 semi-structured interviews will be conducted remotely (via Microsoft Teams or telephone) or in-person at participants’ workplaces, such as company offices or care homes. We aim to interview 10 staff members from VR companies and 10 care home staff. This sample size is well-suited for qualitative research, informed by literature indicating that saturation is often achieved after 9–17 interviews.^44^

Participants will be senior care home staff who have used VR training or staff from companies that provide VR training to care homes, and who are proficient in speaking and reading English, based anywhere in the UK.

We will recruit participants through various channels, including company websites, news articles, snowball sampling (referrals from other participants) and our networks, such as the PPIE group and Greater Manchester Integrated Care Board (GM ICB). Email invites will contain a study summary and the participant information sheet. We will follow-up with participants no more than two times.

#### Procedures

Consent will be obtained in writing or verbally, depending on the interview format. Participants can share their contact information (eg, email address) to receive project updates, including lay summaries and a £20 voucher as a token of appreciation.

The interviews will be semi-structured and audio-recorded for analysis. Each interview will last no more than 1 hour. A bespoke interview schedule will be developed for each set of interviews (VR company staff and care home staff). Both will start by covering employment activities, role and experience with VR. The interviews with senior care staff will explore contextual and organisational issues that must be incorporated into the training package and toolkit, and explore how care homes record data on falls and infections (for phase 2). Interviews with the staff from VR companies will help us understand their cost models and the resources they provide alongside the training to support use and uptake (eg, training guides, pre-training scenarios and structured communications with care homes). If possible, we will seek to obtain documentary evidence of related resources to inform our training materials.

#### Analysis

All data collected will be digitised, anonymised, stored and managed in an NVivo file accessible to the qualitative researchers. The qualitative researchers will analyse the data iteratively and concurrently with data collection, allowing arising topics to be explored in subsequent interviews. As earlier, thematic analysis will be used, following the four steps to integration.^43^

The findings will inform the development of the toolkit, including what form it will take and what the content will be. We will also draw on the responses from participants taking part in the testing phases. Once developed, the toolkit will be tested along with the intervention in the third round of testing.

### Phase 2: pilot study with process evaluation to explore VR training and develop the measures and methods to inform an RCT

We will conduct a mixed-methods pilot cluster RCT with a waitlist control and embedded process evaluation (to address objectives 2–4). There is a 3-month follow-up time point in addition to data collected at baseline and after the intervention (VR training).

The pilot cluster trial will test the feasibility of conducting a larger RCT, focusing on the measures and methods of evaluating the outcomes and reactions to the training (including cyber-sickness and the care home culture). The intervention will be the VR training package plus the implementation toolkit. The control group will not have any intervention but will be a waitlist control. The waitlist control allows the control group to access the training at the end of their follow-up period.

The process evaluation will generate knowledge about VR as a training mechanism in care homes, focusing on the practicality of using VR, contextual considerations and how it might be scaled up in the future.

#### Pilot cluster RCT

##### Setting, participants, recruitment and randomisation

Six care homes in North England will be recruited purposively to reflect the diversity of care homes in the UK. We will seek representation from care homes by different types (nursing, residential or mixed), size (number of beds), ownership (independent vs chain; private, charity or public) and location (deprivation).

We will recruit two small (1–50 beds), two medium-sized (51–100 beds) and two large (101–250 beds) care homes. A total of 80 staff will be recruited: 5 from each small care home, 15 from each medium home and 20 from each large home. The sample size has been selected to allow a sufficient number of repetitions of the protocols to identify any barriers and to permit assessment of feasibility outcomes.

Participants will meet all of the following inclusion criteria to take part:

1. Be an unregistered staff member employed at the care home, providing direct care to residents.
2. Read and write in English.
3. Not have previous health conditions that render them ineligible to use a VR headset due to safety reasons (eg, history of seizures).

Registered staff (eg, nurses), those unable to read/write in English or who do not provide care (eg, domestic staff or chefs) will be excluded. Randomisation will be stratified by the size of the care home, as measured by the number of beds (small (1–50 beds), medium-sized (51–100 beds) and large (101–250 beds)). Each study arm will contain one large, one medium and one small care home. All staff participants at a single site will be recruited before site allocation is revealed to ensure prospective concealment of the randomisation sequence. The randomisation sequence will be created by a study statistician, and each allocation will be revealed only after all staff participants at a site have been unambiguously enrolled in the study.

To reduce participant burden, all activities will be conducted during work hours (with the manager’s support) and on-site at the care homes, in private rooms. This may include an empty resident room, an activities room or a staff room. The researchers will be present on-site to conduct the study, meaning they can be flexible in rescheduling sessions if care work arises.

Staff will be recruited directly via the managers and senior care staff. They will identify all care staff who meet the eligibility criteria and will provide them with a written participant information sheet.

Only the intervention group will receive the VR risk reduction training with the toolkit during the trial period. Outcome data will be compared between groups. At the end of the study period, we will give the three care homes in the control group the opportunity to test the training. This is to encourage their initial participation and ensure that their staff can also benefit from the intervention.

##### Procedures

The research team will take verbal or written consent prior to participation. During the consent process, participants will be asked whether they would like to share their contact information to receive updates on the project (eg, lay summaries). For resident falls and infections data, we will obtain consent at the care home level, as we are not collecting identifiable information.

All staff participants will receive shopping vouchers for their time. They will receive a £20 voucher after the first session and a second £20 voucher at the end of the 3-month follow-up session. Each session should not take more than 1 hour.

Table 2 presents the data we will collect/record using bespoke and validated questionnaires, observations and system data.

**Table 2:** Data to be collected during the pilot cluster RCT

**Baseline**	
*Contextual participant and care home-level data*	
Contextual data	Staff demographics, role and years working in care homes
	Care home size
	Type: residential/nursing
	Indices of Multiple Deprivation score
	Care Quality Commission quality rating and specialities
	Technical infrastructure (eg, availability of WiFi, access to computers/remote devices, etc)
	Previous training delivered and cost model
**Feasibility outcomes**	
Trial feasibility	Recruitment and retention of sites and participants, as well as the number and % of staff trained. Reasons given for declining to participate (if applicable)
Training completion	Number and % of training sessions completed, and time taken
Observations	Qualitative observations (field notes taken by the researchers) on reactions, management policies, practices and care home culture and fidelity with the training schedule (considering the logic model from phase 1) to assess whether activities ran as planned
Cybersickness (post-training)	Validated and existing CSQ-VR^39 40^ to explore adverse effects related to using a VR headset (eg, fatigue or nausea)
**Candidate primary outcomes**	
*Learning impacts (at baseline, after the training for the intervention group and after 3 months post-training)*	
Knowledge	Amend the WHO Hand Hygiene Knowledge Questionnaire for Healthcare workers^53^ and the Singapore Ministry of Health Nursing Clinical Practice Guidelines on Prevention of Falls in Hospitals and Long Term Care Institutions^54^ to align with care home practices
Skill	Hand hygiene will be video-recorded and compared with the WHO six poses.^55^ Participants will identify fall risks in a picture of a simulated room. We will use multiple scenarios which are similar in difficulty to reduce any potential for contamination. The measure will be developed by drawing on the expert knowledge of our research team in falls prevention, using the current evidence base for care homes. When developing this, we will ensure to test the face validity of the measure. The scenarios may draw on the 4 P’s (or similar), which include: Pain: whether the resident is experiencing discomfort which may affect balance or mobility. Personal needs: whether residents need the toilet or other personal care as urgency can lead to rushing and unsafe movements. Position: checking residents are positioned safely—seated, standing or moving in a stable way. This also includes considering circulation or blood pressure changes and whether any equipment they need (eg, Zimmer frames) works and is within reach. Place: checking the care home environment for hazards like rugs, clutter or poor lighting
Self-efficacy	Amend items from our previous study,^23^ as existing task-specific scales are irrelevant to hand hygiene or falls
**Secondary outcomes**	
*Resident impacts (at baseline and after 3 months post-training)*	
Resident impacts	Depending on the findings from phase 1, we will collect data on falls reports and falls-related hospital admissions, emergency department (ED) attendance, GP callouts, outbreaks of infectious diseases and medication changes (antibiotic use) to help determine the feasibility of assessing impacts in a future RCT
*Employment (at baseline and after 3 months)*	
Employment	Staff satisfaction using a validated survey, for example, Job Satisfaction Questionnaire (JS-Q)^45^ or Satisfaction of Employees in Healthcare (SEHC)^46^
	Staff retention and recruitment. Staff illness and any management changes
*Cost data*	
Intervention (and health and social care) costs	Intervention costs will be calculated by micro-costing the resources used during the VR training development, adoption and delivery and split into non-recurrent and recurrent costs. A Client Service Receipt Inventory will record resident falls and infection-related numbers of inpatient hospital days, ED attendances and GP callouts. This data will help determine the cost of healthcare use in the intervention and control group care home residents

CSQ-VR, 6-item Cybersickness in VR Questionnaire; GP, general practitioner; RCT, randomised controlled trial; VR, virtual reality.

We envision the data to be counts of the number of instances of an outcome over the study period. Part of the feasibility study will involve exploring how to collect this data effectively. For example, this could include receiving reports on outcomes from the care home, auditing resident data or asking care homes to provide data into a secure data capture portal (eg, REDCap).

##### Analysis

Where relevant (eg, for the contextual care home-level data, some feasibility outcomes, primary candidate outcomes, resident impacts and staff retention and recruitment data), quantitative data will be analysed using descriptive statistics (eg, mean, sums, SD, percentages). The promise of the intervention will be assessed by comparing hand hygiene and falls skills between the groups, adjusting for clustering and the baseline score using analysis of covariance to increase power. A significance level of 10% will be used.

Analysis of validated surveys will adhere to guidelines. A total score and sub-scores for the nausea, disorientation and oculomotor symptoms will be produced for the CSQ-VR.^39 40^ The sub-scores will be calculated by adding the responses (Likert scale: 1–7) to the two questions related to each symptom. The total score is the sum of the sub-scores. Similarly, for the JS-Q,^45^ a total score and sub-score can be generated by adding the responses (Likert scale 1–5) to each item within the eight domains and calculating a total score, with higher scores indicating better job satisfaction. The Satisfaction of Employees in Healthcare^46^ is analysed by calculating a mean score of the 18 items, with higher scores reflecting higher levels of satisfaction.

Although data on costs and a range of potential outcome measures will be collected, a definitive economic evaluation will not be performed. Summary statistics will be presented for cost and outcomes data, including rates of missing data. These measures will help inform the methods for an economic evaluation of VR training in the future RCT.

Qualitative data gathered through written observations will be analysed using NVivo. The observations will be analysed using a content synthesis approach to help identify commonalities among participant reactions to using VR and the care home culture, while making comparisons to the logic model (phase 1)gives a deeper understanding of whether (and how) the activities ran as planned.

### Process evaluation

Process evaluations should be a collaborative approach, so the PPIE group and relevant stakeholders will be engaged to:

1. Develop and verify the logic model for the intervention. Logic models can prioritise and structure research questions and data collection to help explain how the intervention works to achieve its outcomes or why it does not always work.
2. Refine and prioritise the process evaluation’s primary and secondary research questions. The research questions will build on the literature and previous work, and these might include questions around:
  - Adherence to the intended delivery of the VR training.
  - Adaptations made to deliver the VR training in different settings.
  - What staff groups do or do not engage with the VR training.
  - How the VR training is perceived among different stakeholders.
3. We will refine and test the initial logic model for the different sites to understand similarities and differences in the contexts, relationships and environments that may shape the implementation of the VR training.

Insights from meetings conducted with the PPIE group, combined with data collected in phase 1, will help us understand the implementation of the training in the future.

#### Setting, participants and recruitment

We will select three organisational cases (care homes) for in-depth qualitative investigation through interviews with relevant stakeholders, aiming to explore the adoption, implementation and scale-up of VR training, as well as the impact on those delivering and receiving it, over time. The three care homes will include one of the small, medium and large care homes recruited through the trial.

Between 4 and 7 participants will be interviewed from each care home and will include staff who have taken part in the training. This could include staff from care homes in the intervention group, those in the control group, after having taken part in the training as part of the waitlist control or those involved in organising the sessions (eg, managers or senior carers).

We will purposively sample participants to ensure maximum variation in terms of individual and organisational contexts, considering potential adjustments to doses and configurations of implementation strategies necessitated by specific features of diverse implementation contexts. Recruitment will continue until data saturation is reached, with an estimated sample size of up to 20 participants (aiming for 14 learners and six managers/facilitators).

#### Procedures

The interviews will be conducted on-site at the care homes or remotely via telephone or Microsoft Teams, as convenient to participants. Interview schedules, developed in collaboration with the PPIE group, will be informed by using the NASSS framework^32^ and the CFIR,^33^ to uncover the underlying mechanisms driving the pilot RCT outcomes and to elucidate how the VR training might be adopted, implemented and scaled up. The interviews will be digitally recorded and transcribed verbatim for analysis.

#### Analysis

Transcripts will be uploaded to NVivo. The qualitative dataset will be analysed using thematic analysis, with some codes and categories developed inductively and others derived from NASSS, CFIR and literature on implementing VR training interventions (eg,^27^). Matrix analysis^47^ will be used to compare different respondent groups and organisations. This first phase of analysis will lead to the development of an analytical framework capturing the key determinants and mechanisms of a successful implementation and scale-up process. We will then synthesise the implementation-related insights gained from all research phases using the CFIR and NASSS to identify the ‘core enabling ingredients’ necessary to achieve sustained adoption and scale-up, as well as ‘adjustable elements’ that may vary across different implementation contexts. This blueprint will describe core barriers and enablers encountered, any variability in uptake that influences outcomes, any capacity for local adaptation, and the costs/resources necessary to embed and sustain the intervention in practice.

### Feasibility of conducting a full trial

This pilot study aims to explore VR training and develop the measures and methods to inform an RCT. We will, therefore, use a traffic light system to determine whether a full trial is feasible. Red indicates that it is not feasible (and that significant amendments are needed), orange that some improvements are needed, and green that it is feasible. We will map our findings to the criteria presented in table 3.

**Table 3:** Pilot progression criteria to determine the feasibility of proceeding with a full-scale randomised controlled trial

Criteria	Interpretation
Care home sites recruited/opened	Red: 0–3 care homes (0%–50%) Orange: 4–5 care homes Green: 6 care homes (100%)
Willingness of care homes to be randomised	Red: 0–3 care homes (0%–50%) Orange: 4–5 care homes Green: 6 care homes (100%)
Retention of care homes at 3 months	Red: 0–3 care homes (0%–50%) Orange: 4–5 care homes Green: 6 care homes (100%)
Training completion (participants)	Red: 60%–74% Orange: 75%–99% Green: 100%
Retention of participants at 3 months	Red: Retain 60%–74% Orange: Retain 75%–79% Green: Retain ≥80%
Completeness of infections and falls data of residents at 3 months	Red: 60%–74% of residents Orange: 75%–79% Green: ≥80%

## Patient and public involvement and engagement

The project will be conducted with input from care home stakeholders, including several authors (JC, CC and VE), who contributed to the funding application and protocol and represent resident relatives and care home staff. The PPIE advisory group has now been broadened to include several care home managers and visitors to care homes. Contributors involved in direct resident care will provide insight into training delivery (and content) and the acceptability of VR technology in education for staff. Additionally, the PPIE group will also assist in recruiting participants, as buy-in from managers and staff is crucial.

The PPIE activities will be structured in accordance with the NIHR UK Standards for Public Involvement.^48^ The PPIE group will help design the training and toolkit, give insights on implementation, oversee the methods, review resources for readability, support recruitment, write lay summaries and coauthor publications. They will also be crucial to informing our wider dissemination plan and helping ensure the project has reach and impact. This is integral to the project due to the future impact on residents and workforce diversity. Participant resources must, therefore, be presented in an accessible manner to ensure inclusive recruitment. PPIE contributors will be given training by the research team to support skills and confidence to contribute meaningfully. We will use the NIHR INCLUDE framework^49^ to guide our PPIE work and participant recruitment, including the number of collaborators and representation (considering gender, age, ethnicity and work experience).

We have also linked in with the Adult Social Care Transformation Team, GM Integrated Care Partnership (ICP), who have identified care homes that want to be involved in the study. The GM Social Care Academy (part of the ICP) has also contributed to this protocol and is eager to support recruitment. Additionally, we have links outside of GM, including ICB links in Yorkshire and Humber, as well as care home networks in Merseyside and Cheshire. The team also has experience in working with the ENRICH network of research-ready care homes.

## Ethical considerations and dissemination

###  Ethical approvals, considerations and mitigation strategies

The project will comply with ethical principles outlined in the Declaration of Helsinki.^50^ Two pathways for ethical approval will be taken due to different data being collected. The University of Manchester’s Proportionate Research Ethics Committee has approved phase 1 activities (Reference: 2025-24416-44642), and approval will be sought for phase 2 by the NHS REC through the Social Care Ethics Committee at the end of 2026/early 2027.

There are few risks and burdens to participating in this research, with the main burden being the time spent by participants during data collection. We will value participants’ time by conducting activities during work hours and compensating them with vouchers. Based on our previous studies, we have identified potential risks that may affect participants and researchers when conducting VR research in care homes (see table 4).

**Table 4:** Ethical considerations and key mitigation strategies

Ethical considerations	Key mitigation strategy
Participant safety (harm)—experiencing cybersickness when using the VR headset	Participants will be provided with information at the beginning of using the VR training regarding symptoms of cybersickness, and if they feel uncomfortable at any time, we will stop the VR module, assist them in removing the headset and give them time to recover. Cybersickness symptoms cease promptly when the headset is removed We will use a variety of different headsets during phase 1 to identify if there is one headset that minimises cybersickness We have included a validated measure of cybersickness as part of our study outcome measures in phase 2 (the CSQ-VR)^39 40^
Participant safety (harm)—movement which has the potential for harming oneself on furniture	We will identify a space in each care home for data collection, which enables some degree of movement by participants We will remove all loose furniture prior to data collection starting and will have a researcher present to prevent participants from coming to harm Most VR headsets prompt users to create a boundary (ie, a safe area to use VR) and, when nearing this boundary, will automatically alert users and the headset camera will turn on, so the user will be able to see their physical position outside of the VR training
Researcher safety (harm)—lone working and travelling to unknown areas	Appropriate protocols from the University of Manchester will be followed to ensure checking in before and after data collection
Informed consent	We will strive to produce care staff-facing research materials in clear, lay language, as appropriate for individuals with limited literacy skills or who speak English as a second language. These will be sense-checked by the PPIE advisory group and informed by the Flesch-Kincaid readability metrics (aiming to score 60 or higher on the Reading Ease Score) Acknowledging that some people have not yet experienced VR, we will include photographs and a description of the technology in the information sheet
Coercion—power imbalance	Staff will be assured that they do not have to participate, and that non-participation will not impact their employment We will reassure all participants (and residents/carers) that no personally identifiable information will be published We will assure staff that their employment satisfaction data and behaviours related to hand hygiene and falls prevention will not be shared with their employer

CSQ-VR, 6-item Cybersickness in VR Questionnaire; PPIE, patient and public involvement and engagement; VR, virtual reality.

### Outputs and dissemination plan

We will work in collaboration with our PPIE contributors and stakeholders (eg, ICBs and care networks) to ensure we develop materials and plan dissemination activities that have the potential to reach the wider care home sector. We have used the 7Cs approach to guide our plan for impact,^51^ where we have identified the context, communities, constituencies and challenges we are addressing with the study. While we have some initial plans for producing outputs from our study and its wider dissemination, this will be revisited and developed throughout the study. We want to ensure that we are developing materials to communicate the findings from the study using appropriate methods for different audiences and the message we wish to communicate, and that we have appropriate measures for demonstrating that we have reached the desired audiences.

Outputs will include a user-friendly and acceptable VR risk reduction training programme for care homes, accompanied by an implementation toolkit adaptable for other VR training in social care settings. We will co-produce these materials with study participants and our PPIE group, and develop communication materials (eg, training overviews, infographics, YouTube videos) to help support the training tools.

Findings will be disseminated both nationally and internationally. They will be presented at relevant conferences and events, which are attended by managers, commissioners and resident relatives and published in journals. Lay summaries will be co-created with our PPIE advisory group, and additional dissemination methods will be co-developed to broaden reach. The lay summaries will be distributed to all interested participants and care home managers who have supported the project. A short report will be disseminated through the National Care Forum, reaching out to care homes across the country at the end of phases 1 and 2. A website for the project will be developed and will be updated on a regular basis with updates on research progression.

## Discussion

Motivated by the need to improve and standardise risk reduction training for unregistered care home staff in the UK, the VR-CARE project initially focuses on hand hygiene and falls prevention modules—both critical yet non-mandatory areas of training in care homes.^4 5^

The project aims to address key unanswered questions regarding the effectiveness, benefits to residents and cost-delivery models of VR-based training for care home staff. Its mixed-method design will provide a comprehensive understanding of several factors influencing training success, including iterative development, usability testing, qualitative insights into implementation, cost implications and measurable impacts on both staff and residents. The inclusion of a 3-month follow-up will also enable exploration of sustained behavioural changes (eg, skills retention) and potential effects on residents, in relation to infections and falls. Together, these priorities will generate crucial evidence on both impact and implementation—knowledge essential for supporting the wider adoption and scale-up of VR training in UK care homes. Moving away from outcomes beyond satisfaction will also ensure that VR is adopted as a validated intervention, rather than novelty, with economic evaluations especially being critical for decision-makers when determining whether VR is worth investing in as a training tool.^52^

Limitations of the project include the limited geographic scope, which may affect transferability to other areas and countries. This is because regional workforce characteristics, organisational cultures and digital infrastructure specific to the North of England may be reflected in the findings. Additionally, it is expected that some inherent technical barriers, such as cybersickness, digital literacy (and confidence) and Internet access may limit engagement, satisfaction or fidelity of delivery. These barriers are difficult to overcome, but are important to understand when assessing the feasibility of delivering VR training for any group of learners, beyond care homes.
