# Supplementary material for: VR-CARE: a protocol for a mixed-methods study and pilot trial with embedded process evaluation to develop and evaluate virtual reality training for risk reduction in care homes
Source: BMJ Open. 2026 Mar 9;16(3):e116603. doi: 10.1136/bmjopen-2026-116603 (PMC12983739; doi:10.1136/bmjopen-2026-116603)
Supplement: Supplementary file 1 [file bmjopen-2026-116603.reviewer_comments.pdf]

PEER REVIEW HISTORY

BMJ Open publishes all reviews undertaken for accepted manuscripts. Reviewers are asked to complete a checklist review form and are provided with free text boxes to elaborate on their assessment. These free text comments are reproduced below.

ARTICLE DETAILS

Title (Provisional)

VR-CARE: A protocol for a mixed-methods study and pilot trial with embedded process evaluation to develop and evaluate virtual reality training for risk reduction in care homes

Authors

Gasteiger, Norina; Ford, Claire R; Hawley-Hague, Helen; Wilkinson, Jack; Jones, Debra; Whittaker, William; Ullah, Akbar; Kislov, Roman; Stanmore, Emma; Laverty, Louise; Chantrell, Joan; Callaghan, Claire; Edmondson, Victoria; Dowding, Dawn

---

VERSION 1 - AUTHOR RESPONSE

This article was not externally reviewed at BMJ Open. Protocols that have been independently assessed prior to submission to BMJ Open are usually fast-tracked to publication on the grounds that further substantial changes will not be possible. This independent assessment will usually be external, independent review for both a substantial grant award from a non-commercial or government funder and ethics approval.
